# Research on a method for early diagnosis and progression prediction of cervical cancer based on imaging omics and molecular omics

**DOI:** 10.3389/fonc.2025.1670852

**Published:** 2026-02-10

**Authors:** Fengdan Sun, Xian Ge, Luohui Deng, Yeman Wang, Hongbo Wu, Ling Wang, Qingling Ren

**Affiliations:** 1 Affiliated Hospital of Nanjing University of Chinese Medicine, Nanjing, Jiangsu, China; 2 The Chinese Clinical Medicine Innovation Center of Obstetrics, Gynecology, and Reproduction in Jiangsu Province, Nanjing, Jiangsu, China; 3 Suzhou Traditional Chinese Medicine (TCM) Hospital Affiliated to Nanjing University of Chinese Medicine, Suzhou, Jiangsu, China

**Keywords:** clinical decision support, healthcare informatics, multimodal data integration, transformer-based diagnostic model, uncertainty calibration

## Abstract

**Introduction:**

In response to the increasing demand for clinically interpretable and reliable diagnostic tools in medical informatics, this study introduces a novel computational framework for the early diagnosis and progression prediction of complex diseases, grounded in the integration of imaging omics and molecular omics. Aligning with the research scope of advanced data-driven solutions in computer science for healthcare applications, this work targets key challenges in multimodal biomedical data analysis, including feature heterogeneity, data imbalance, and diagnostic uncertainty. Traditional diagnostic models, often relying on single-modal data or shallow machine learning methods, struggle to capture non-linear dependencies across heterogeneous feature spaces and lack reliable uncertainty quantification for clinical decision-making, resulting in limited generalization and interpretability on real-world, imbalanced clinical datasets.

**Methods:**

To address these limitations, we propose a transformer-based diagnostic encoder, CervixFormer, coupled with a Domain-Aware Calibration Strategy (DACS). CervixFormer leverages hierarchical attention mechanisms and cross-modality feature fusion to extract comprehensive diagnostic representations from high-dimensional imaging and omics data. The framework incorporates imbalance-aware embedding layers and stochastic uncertainty modeling to enhance robustness against noisy and unevenly distributed samples. Furthermore, DACS introduces domain-guided probabilistic recalibration by integrating clinical priors and uncertainty estimates, optimizing the alignment between predicted confidence and true diagnostic risk.

**Results and discussion:**

Extensive experiments conducted on large-scale multimodal datasets demonstrate that the proposed framework significantly outperforms conventional machine learning and deep learning baselines in terms of diagnostic accuracy, robustness, and calibration reliability. The results indicate substantial improvements in handling data imbalance and uncertainty, while maintaining strong predictive performance across heterogeneous modalities. These findings highlight the effectiveness of combining transformer-based multimodal representation learning with domain-aware uncertainty calibration for clinical diagnostics. The proposed framework not only enhances predictive accuracy but also improves confidence reliability and interpretability, which are critical for real-world clinical decision support. Overall, this study underscores the potential of advanced multimodal learning architectures to advance data-driven healthcare applications and provides a promising direction for reliable and clinically applicable diagnostic systems.

## 1 Introduction

Cervical cancer continues to pose significant health risks worldwide, with early detection and accurate progression prediction still presenting major clinical hurdles, especially in resource-limited settings Singh et al. (1). Moreover, traditional cytology and HPV testing, while reducing incidence, often lack sufficient sensitivity and specificity for individualized prognostication Lu et al. (2). In addition, emerging insights from molecular and imaging data suggest that multimodal integration could bridge this gap, enabling personalized risk stratification. Therefore, developing a unified framework that leverages both imaging omics (radiomics features from MRI, PET/CT) and molecular omics (genomics, transcriptomics, proteomics) is not only timely but necessary to enhance early diagnosis and monitor disease evolution with greater precision and clinical impact Ding et al. (3).

Initial efforts in diagnostic modeling primarily relied on predefined rules and domain-specific mappings. These systems often linked observed patterns—such as radiologic textures or specific genetic mutations—to manually curated disease indicators Chakraborty et al. (4). While this approach laid foundational clinical associations Newaz et al. (5), its dependence on handcrafted logic and human annotation restricted its scalability. The rigidity of such systems also hindered adaptation when encountering patient variability or unforeseen data distributions Nithya and Ilango (6).

Subsequent developments introduced algorithms capable of automatically learning statistical relationships from selected features Tseng et al. (7). These techniques, including linear and nonlinear classifiers, utilized curated descriptors from imaging and genomic datasets to improve classification performance. Strategies like ensemble learning and dimensionality reduction increased model generalizability Lee et al. (8). However, these methods still required substantial manual input to select and combine features from different data sources, limiting their ability to model the full complexity of tumor biology and microenvironmental heterogeneity Qureshi et al. (9).

More recent advances have shifted toward unified architectures that learn hierarchical representations directly from raw imaging and molecular inputs Ali et al. (10). These models can simultaneously capture morphological structures, molecular interactions, and their cross-modal correlations. Techniques such as joint embedding and multi-level attention have demonstrated promise in uncovering latent patterns for early lesion recognition and disease trajectory modeling Matsuo et al. (11). Nonetheless, challenges remain in ensuring interpretability, managing data imbalance, and aligning model outputs with clinically meaningful decision thresholds—especially in real-world, multi-institutional settings Sarwar et al. (12).

To further address the weaknesses of classical methods, recent advances have embraced deep learning and pre-trained models that jointly model imaging omics and molecular omics in an end-to-end framework. Convolutional neural networks extract hierarchical image features, while graph neural networks or transformer architectures can encode molecular interaction networks. Pre-training on large public datasets or using self-supervised methods enables feature transfer across institutions. These deep models demonstrate superior accuracy in predicting early lesions and forecasting progression, and they facilitate multimodal fusion. However, they face challenges in requiring large labeled datasets, providing clinical interpretability, and ensuring robustness to domain shift.

A number of notable strengths distinguish the proposed approach, including:

- A fusion module is proposed to unify radiomics and multi-omics encoding. Cross-layer attention mechanisms enable feature interaction, enhancing multimodal representation capacity. Based on this design, the model generalizes without requiring additional modules.
- The method is applicable across diverse scenarios (MRI, PET/CT, gene expression, proteomics). It employs lightweight pretraining and module-sharing strategies, significantly reducing computational costs and adapting well to small-sample settings.
- On multi-center datasets, diagnostic AUC is improved to 0.92, and progression prediction accuracy increases by 15%. Attention visualization verifies the biological significance of key imaging and molecular biomarkers discovered by the model, improving clinical interpretability and trust.

## 2 Related work

### 2.1 Radiomic feature extraction and analysis

In recent years, the field of radiomics has emerged as a powerful modality for noninvasive early diagnosis and progression prediction of cervical cancer Lucia et al. (13). Radiomic analysis begins with precise segmentation of medical imaging data—such as MRI, CT, and PET scans—to delineate tumor regions, using both manual expert delineation and automated segmentation techniques driven by deep learning. Following segmentation, a high-dimensional feature extraction pipeline is applied to quantify tumor heterogeneity through texture, shape, intensity, and wavelet-derived metrics. Spatial intensity patterns are effectively represented by properties such as entropy, contrast, and correlation derived from gray-level co-occurrence matrices, which describe how pixel values relate across local neighborhoods; shape features quantify morphological characteristics such as compactness, sphericity, and surface-to-volume ratio; intensity features record first-order statistics like mean, variance, skewness, and kurtosis; and wavelet features reveal multiscale patterns. The extracted features require thorough preprocessing, including standardization, normalization, and dimensionality reduction through methods such as principal component analysis or LASSO, to address redundancy and minimize the risk of overfitting Zhang et al. (14). Feature selection is optimized by supervised learning methods such as recursive elimination and stability selection, ensuring identification of the most reproducible and biologically meaningful metrics. Once selected, these radiomic signatures are incorporated into machine learning models designed to distinguish between benign and malignant lesions while enabling stage stratification of cervical cancer. For progression prediction, these models leverage complex patterns within the features to anticipate disease advancement with greater accuracy Yang et al. (15), longitudinal imaging data are analyzed to extract delta-radiomic features (changes in radiomic attributes over time), capturing subtle temporal alterations associated with tumor aggressiveness or therapeutic response. Multimodal radiomic signatures further enhance predictive accuracy by fusing imaging modalities—such as combining T2-weighted MRI and diffusion-weighted imaging—thus capturing complementary structural and functional tumor characteristics. Validation of radiomic models is conducted through rigorous internal cross-validation and independent multicenter external validation cohorts to assess generalizability Vickers and Elkin (16). Reproducibility analyses, intraclass correlation coefficients, and test–retest studies ensure feature stability. Integration of radiomic signatures into nomograms or risk scores alongside clinical variables (age, tumor grade, HPV status) yields predictive tools with improved discrimination power for early-stage diagnosis and progression 106 forecasting Law et al. (17).

### 2.2 Molecular omics biomarker discovery

Molecular omics encompasses high-throughput profiling technologies—such as genomics, transcriptomics, epigenomics, proteomics, and metabolomics—aimed at identifying molecular biomarkers for early cervical cancer detection and disease progression prediction. Genomic analyses employ next-generation sequencing to detect somatic mutations, copy number variations, and structural rearrangements in tumor DNA sampled via biopsy or liquid biopsy (circulating tumor DNA). Recurrent alterations in genes such as PIK3CA, TP53, and EP300 provide insight into oncogenic pathways Martin et al. (18). Transcriptomic profiling (RNA-seq) reveals differential gene expression signatures between precancerous lesions, early-stage disease, and advanced carcinoma; noncoding RNA analysis—including microRNAs (miR-21, miR-34a) and long noncoding RNAs—adds prognostic value by reflecting regulatory networks linked to tumor invasiveness. Epigenomic assays characterize DNA methylation patterns at promoter CpG islands; hypermethylation of tumor suppressor genes (DAPK1, RARB) and global hypomethylation are predictive of transformation. Proteomic approaches—mass spectrometry–based or antibody arrays—quantify protein expression and post-translational modifications; overexpression of proteins like MMP-9, VEGF, and cytokeratins correlates with tumor angiogenesis and metastasis risk. Metabolomic profiling via NMR or LC-MS identifies altered metabolic pathways (choline metabolism, glycolysis) Biewenga et al. (19), whose metabolites serve as diagnostic signatures. Integrative analysis pipelines combine these multi-omic layers, using unsupervised clustering and network-based approaches to identify molecular subtypes and biomarkers that predict disease onset and progression. Statistical methods (Cox regression, LASSO-Cox) evaluate association between candidate biomarkers and clinical outcomes Gadducci et al. (20). Development of multi-omics risk panels—combining genomic mutations, methylation scores, protein markers, and metabolite concentrations—enhances detection sensitivity and specificity compared to single-omic assays. Validation in prospective cohort studies and platform reproducibility studies—such as technical replicates and independent sample sets—is critical Wanram et al. (21). Integration of molecular biomarker panels with imaging omics signatures through machine learning–based data fusion improves robustness and predictive power, enabling biomarker-enriched models capable of early detection and accurate projection of progression risk.

### 2.3 Imaging–molecular multiomics integration

Integration of imaging omics and molecular omics, termed radiogenomics or multiomics, represents the frontier of precision diagnostics in cervical cancer Yen et al. (22). This approach leverages the complementary strengths of noninvasive imaging and molecular profiling to improve early diagnosis and prognosis prediction. The first step involves parallel acquisition of imaging data and molecular profiles in the same patient cohort. Imaging omics data—radiomic features—are spatially mapped to correlate with tumor regions subjected to molecular assays, enabling voxelwise or region-of-interest radiogenomic mapping. Molecular datasets include genomic mutation calls Galbán et al. (23), transcriptomic expression values, methylation status, proteomic intensities, and metabolite levels. Multimodal data fusion is achieved through feature-level integration—concatenating radiomic and molecular variables—and decision-level integration, where separate predictive models for each modality are combined via ensemble methods. Advanced integrative modeling utilizes sophisticated architectures like multimodal autoencoders to learn joint latent representations that encapsulate shared information between imaging and molecular data. These approaches enable unified analysis across diverse modalities, enhancing the understanding of underlying biological patterns. Graph-based multilayer networks and canonical correlation analysis also facilitate discovery of cross-modal associations. These integrative models are trained to predict diagnostic labels (presence of early-stage carcinoma) and prognostic endpoints (progression-free survival, metastasis risk) using supervised learning methods optimized with regularization techniques to handle high dimensionality and limited sample sizes Yen and Lai (24). Explainability strategies—such as SHAP values or attention maps—are essential to identify imaging features linked to specific molecular pathways, aiding biological interpretability and clinical trust. Robust validation on external multi-center cohorts is required to confirm generalizability. Successful imaging–molecular models have demonstrated predictive AUC improvements over single-modality models, and have linked imaging phenotypes (heterogeneous texture, necrotic zones) to molecular hallmarks (TP53 mutation, immune pathway activation). Integration into clinical workflows involves constructing multimodal nomograms and risk calculators, enabling clinicians to stratify patients at pre-invasive stages and adapt treatment decisions Gurcan et al. (25). Ultimately, imaging–molecular multiomics integration offers a path toward personalized early detection and tailored monitoring strategies for cervical cancer patients.

## 3 Method

### 3.1 Overview

Cervical cancer remains one of the most prevalent malignant tumors affecting women globally, posing significant health challenges, especially in low- and middle-income countries. Despite continuous advancements in screening programs and vaccination strategies, the burden of cervical cancer persists, demanding innovative computational approaches to facilitate early detection, precise diagnosis, and robust prognosis prediction.

In response to this critical demand, our research introduces a novel computational framework that addresses the limitations of existing diagnostic systems by leveraging recent breakthroughs in machine learning and medical image analysis. Cervical cancer diagnosis generally relies on a combination of cytological, histopathological, and molecular data—each presenting challenges such as high dimensionality, heterogeneous feature spaces, and substantial inter-patient variability. These data-specific challenges often lead to issues like class imbalance, complex feature entanglement, and ambiguous decision boundaries, which standard models fail to resolve effectively. Section 3.3 formally defines the cervical cancer diagnosis problem using precise mathematical notation. This includes the representation of multimodal inputs, the structure of diagnostic labels, and a detailed discussion on the assumptions underlying current diagnostic approaches. The foundation set in this section supports the modeling strategies that follow. Subsequently, Section 3.4 details our proposed model, CervixFormer, a transformer-based framework tailored for cervical cancer analysis. CervixFormer employs multi-head attention to extract both global and local features, integrates hierarchical representation learning, and introduces a dual-branch architecture to manage morphological and molecular inputs simultaneously. By embedding domain-specific features and using position-aware encoding schemes suited for irregular medical data, the model effectively addresses the challenges of feature sparsity and class imbalance.

To highlight the distinctions of our approach, we elaborate on how each module in CervixFormer improves upon prior work. First, unlike traditional early-fusion or late-fusion strategies which treat modality features independently or concatenate them without semantic interaction Zhang et al. (26), our multimodal attention fusion explicitly models cross-modality interactions through hierarchical attention and cross-attention mechanisms, allowing dynamic inter-modal dependencies to emerge. Second, existing methods typically address class imbalance via data resampling or weighted loss functions, which often overlook feature-level learning dynamics. In contrast, our imbalance-aware scaling mechanism modulates the feature space adaptively by integrating both class frequency and instance difficulty into the embedding process. This ensures balanced representation learning without distorting feature distributions. Third, while uncertainty modeling is rarely integrated into multimodal cervical cancer diagnostics, we introduce a principled uncertainty-aware learning mechanism that captures epistemic uncertainty using Monte Carlo dropout and predictive variance, inspired by works in Bayesian deep learning. Moreover, the Domain-Aware Calibration Strategy (DACS) aligns predictive confidence with clinical thresholds, improving both reliability and interpretability—two aspects underrepresented in prior transformer-based models. These design innovations collectively ensure that CervixFormer achieves robust generalization and clinically meaningful predictions.

To further enhance prediction quality and trustworthiness, we propose in Section 3.5 a decision mechanism named Domain-Aware Calibration Strategy (DACS). DACS improves the interpretability and robustness of the model’s outputs through probabilistic score adjustment, guided by clinical priors and intra-class feature dynamics. It adapts the model’s output confidence in alignment with medical ontologies and established risk assessment practices, thereby bridging the gap between algorithmic predictions and clinical decision thresholds. Together, CervixFormer and DACS form a comprehensive framework that addresses longstanding pain points in automated cervical cancer diagnosis, including feature complexity, diagnostic uncertainty, and clinical integration. Through a combination of rigorous mathematical modeling, architectural innovation, and strategy design, our framework aims to deliver a clinically aligned, performance-optimized, and interpretable diagnostic solution.

We clearly define the classification, segmentation, and progression prediction tasks, specifying which datasets support each. Evaluation metrics for classification (Accuracy, AUC, F1), segmentation (Dice, Jaccard, pixel accuracy), and survival prediction (C-index, IBS) are introduced here for clarity.

Each dataset’s preprocessing pipeline is described separately. For example, SIPaKMeD images undergo rotation-based augmentation, Cx22 features are normalized with z-score, and missing values are handled using imputation. Patch-based extraction is only applied to Pap Smear and CERVIX93.

We consolidate training hyperparameters, backbone choices, and fusion strategies. Dataset-specific training regimes (e.g., segmentation vs classification) are explicitly separated. Batch size, learning rates, and training duration are reported independently for each setup.

### 3.2 Datasets

We utilize four datasets in this study: SIPaKMeD (single-cell classification), Cx22 (clinical tabular classification), Pap Smear (whole-slide classification and segmentation), and CERVIX93 (high-resolution segmentation). Each dataset is described with respect to its task, preprocessing steps, and annotations. For instance, Pap Smear and CERVIX93 are only used for segmentation and require 512×512 patches, while SIPaKMeD images are resized to 224×224 for classification. The SIPaKMeD Al-asbaily et al. (27). Dataset is a high-quality collection designed for the classification of cervical cell images, sourced from Pap smear tests. It consists of over 9,000 single-cell images manually extracted and labeled into five different cell types, including superficial-intermediate, parabasal, and metaplastic cells. The dataset provides pixel- level annotations and standardized segmentation masks, making it highly suitable for both detection and classification tasks. Its balanced structure and expert-verified labels contribute to reliable model training and benchmarking in cervical cytology. SIPaKMeD is widely used in developing robust algorithms that require fine-grained differentiation between morphologically similar cell types, thereby playing a key role in advancing automated cervical cancer screening systems. The Cx22 Liu et al. (28) Dataset is a more recent benchmark focused on real-world cervical cancer screening scenarios. It comprises whole-slide images and region-level annotations derived from a diverse patient population across multiple clinical centers. Unlike traditional cell-level datasets, Cx22 emphasizes the heterogeneity of clinical samples and includes a broader range of visual patterns, such as inflammation and artifacts. This complexity makes it ideal for evaluating generalization in end-to-end diagnostic models. The dataset also includes patient metadata, enabling multimodal studies that combine imaging with demographic or clinical information. Cx22 thus supports the development of holistic diagnostic frameworks that reflect the challenges encountered in routine cytopathology workflows. The Pap Smear Hussain et al. (29) Dataset is a foundational resource in cervical cytology research, containing microscopic images of Pap smear slides labeled into normal and abnormal categories. It offers a balanced representation of both low-grade and high-grade lesions, providing an effective training base for models targeting binary or multi-class classification. The dataset has been instrumental in early developments of computer-aided diagnosis systems due to its accessibility and interpretability. Each image is accompanied by concise clinical context, helping algorithms to learn discriminative features under guided supervision. As a result, the Pap Smear Dataset remains a go-to choice for researchers aiming to validate lightweight and interpretable models in the context of cervical cancer screening. The CERVIX93 Russo et al. (30) Dataset is a curated compilation of high-resolution cervical cell images acquired under consistent imaging conditions to ensure data uniformity. It includes expertly annotated images representing multiple diagnostic categories, such as normal, inflammation, and pre-cancerous stages. CERVIX93 is particularly valuable for tasks that require fine-tuned morphological analysis, including feature extraction and unsupervised representation learning. The dataset’s structure supports rigorous cross-validation and comparative evaluation of deep learning models. Because of its controlled acquisition protocol and detailed labeling, CERVIX93 is frequently adopted in studies focused on reproducibility and model robustness across clinical environments.

Among the four datasets used in this study, only Cx22 contains both imaging features and molecular omics information such as genomics and proteomics. Therefore, the multimodal fusion architecture of CervixFormer, including cross-modal attention mechanisms and DACS-based calibration, was fully enabled for the Cx22 experiments. The molecular components in Cx22 consist of structured clinical variables (e.g., age, grade, race), genomic indicators, and other molecular measurements, which were normalized and embedded into the modality-specific branches. In contrast, the SIPaKMeD, Pap Smear, and CERVIX93 datasets are purely imaging-based and do not include molecular omics data. For these datasets, the CervixFormer framework was operated in an imaging-only configuration, with the multimodal components deactivated. This ensures fair comparison with prior image-based models and isolates the impact of the visual encoder and spatial attention mechanisms. We have now clarified this configuration in the revised manuscript to avoid ambiguity. While the paper presents CervixFormer as a multimodal architecture, it is designed to operate adaptively depending on the modality availability in each dataset. The flexibility of our design allows ablation across different modality settings and ensures that the multimodal claims are only applied to the appropriate experimental contexts. This clarification supports transparency, reproducibility, and a more accurate interpretation of the role of molecular omics integration in our evaluation.

### 3.3 Preliminaries

To formally define the computational problem of cervical cancer diagnosis, we first construct the underlying mathematical framework that governs data representation, model learning, and diagnostic complexity. Let $X\subseteq {\mathbb{R}}^{d}$ be the *d*-dimensional feature space in which each instance x∈X encodes multimodal information from various clinical sources, including cytological patterns, histopathological textures, genomic alterations, proteomic levels, and demographic indicators. The corresponding output label space is defined as $Y=\{y_{1},y_{2},\ldots ,y_{K}\}$, where *K* denotes the number of clinically distinct diagnostic classes. Our objective is to learn a mapping function *f_θ_* : X → ${\text{Δ}}^{K-1}$, where *θ* represents model parameters and ${\text{Δ}}^{K-1}$ is the (*K* − 1)-simplex that ensures probabilistic class predictions. The model produces a probability vector Equation 1:

(1) $$f_{\theta }\text{x}=[p_{1}(\text{x}),p_{2}(\text{x}),\ldots ,p_{K}(\text{x})],\;\sum_{k=1}^{K}p_{k}(\text{x})=1,$$

and the predicted label is determined via maximum likelihood Equation 2:

(2) $$\hat{y}=\text{arg }\underset{k}{\max}\;pk\left(\text{x}\right).$$

However, a major issue in real-world cervical cancer datasets is severe class imbalance, where the frequency of negative cases dramatically outweighs that of positive or high-risk cases. If *N_k_* denotes the number of samples in class *k*, then the imbalance ratio is expressed as Equation 3:

(3) $$\rho =\frac{\max_{k}N_{k}}{\min_{k}N_{k}}.$$

This imbalance often leads to skewed learning dynamics and suboptimal decision boundaries. Given a dataset $D={\left\{\left({\text{x}}_{i},y_{i}\right)\right\}}_{i=1}^{N}$, drawn from the empirical distribution ${\hat{P}}_{D}\left(\text{x},y\right)$ approximating the true data distribution $P\left(\text{x},y\right)$, the learning objective becomes the minimization of expected classification risk Equation 4:

(4) $$R\left(\theta \right)=E_{\left(\text{x},y\right)∼P}\left[\ell (f_{\theta }(\text{x}),y)\right],$$

where *ℓ* is the loss function, typically cross-entropy. Since *P* is unknown, empirical risk minimization (ERM) is used Equation 5:

(5) $$\hat{R}(\theta )=\frac{1}{N}\sum_{i=1}^{N}\ell \left(f_{\theta }{(}_{xi}),y_{i}\right).$$

Cervical cancer data often spans multiple modalities. Let each input vector be structured as $\text{x}=[{\text{x}}^{\left(1\right)}),\ldots ,{\text{x}}^{\left(M\right)}]$, where each modality ${\text{x}}^{\left(m\right)}\in {\mathbb{R}}^{d_{m}}$ and $\sum_{m=1}^{M}d_{m}=d$. These form a multimodal product space Equation 6:

(6) $$X=X^{\left(1\right)}\times X^{\left(2\right)}\times \ldots \times X^{\left(M\right)}.$$

To integrate these heterogeneous inputs, a fusion function *ϕ* is introduced to project the modalities into a unified latent space Equation 7:

(7) $$\text{h}=\phi \left({\text{x}}^{\left(1\right)},\ldots ,{\text{x}}^{\left(M\right)}\right),\;\;\text{h}\in {\mathbb{R}}^{h}.$$

Another dimension of complexity comes from longitudinal follow-up data. For patient *i*, observations across time points $t_{j}\in T$ form a sequence ${\text{X}}_{i}^{\left(t\right)}=\left[{\text{x}}_{i}^{\left(t_{1}\right)},\ldots ,{\text{x}}_{i}^{\left(t_{\left|T_{i}\right|}\right)}\right]$, which is aggregated using a function *ψ*Equation 8:

(8) $$g_{i}=\psi ({\text{X}}_{i}^{\left(t\right)}),$$

resulting in a time-aware patient representation. Moreover, diagnostic labels are susceptible to noise. Let $\eta \in {\mathbb{R}}^{K\times K}$ be the label transition matrix capturing mislabeling probabilities, such that Equation 9:

(9) $$\text{Pr}(y_{i}^{obs}=k\;|\;y_{i}^{true}=l)=\eta_{lk}.$$

Many real-world datasets contain missing entries. For a sample *i*, let $M_{i}$ be the index set of missing features. The observed vector is Equation 10:

(10) $${\tilde{\text{x}}}_{i}=\{\begin{array}{ll} x_{ij}, & j\notin M_{i}\\ \text{NaN}, & j\in M_{i} \end{array}$$

Taking all these challenges into account, the overall learning objective incorporates additional regularization and robustness terms Equation 11:

(11) $$\underset{\theta }{\min}\hat{R}\left(\theta \right)+\lambda \text{Ω}\left(\theta \right)+\gamma L_{\text{imbalance}}+\beta L_{\text{uncertainty}}.$$

Here, Ω(*θ*) represents a weight regularizer, $L_{\text{imbalance}}$ penalizes biased learning, and $L_{\text{uncertainty}}$ models noise-aware prediction risk. This comprehensive formulation establishes the theoretical groundwork necessary for the subsequent architectural and strategic innovations.

### 3.4 CervixFormer

**Figure 1:**
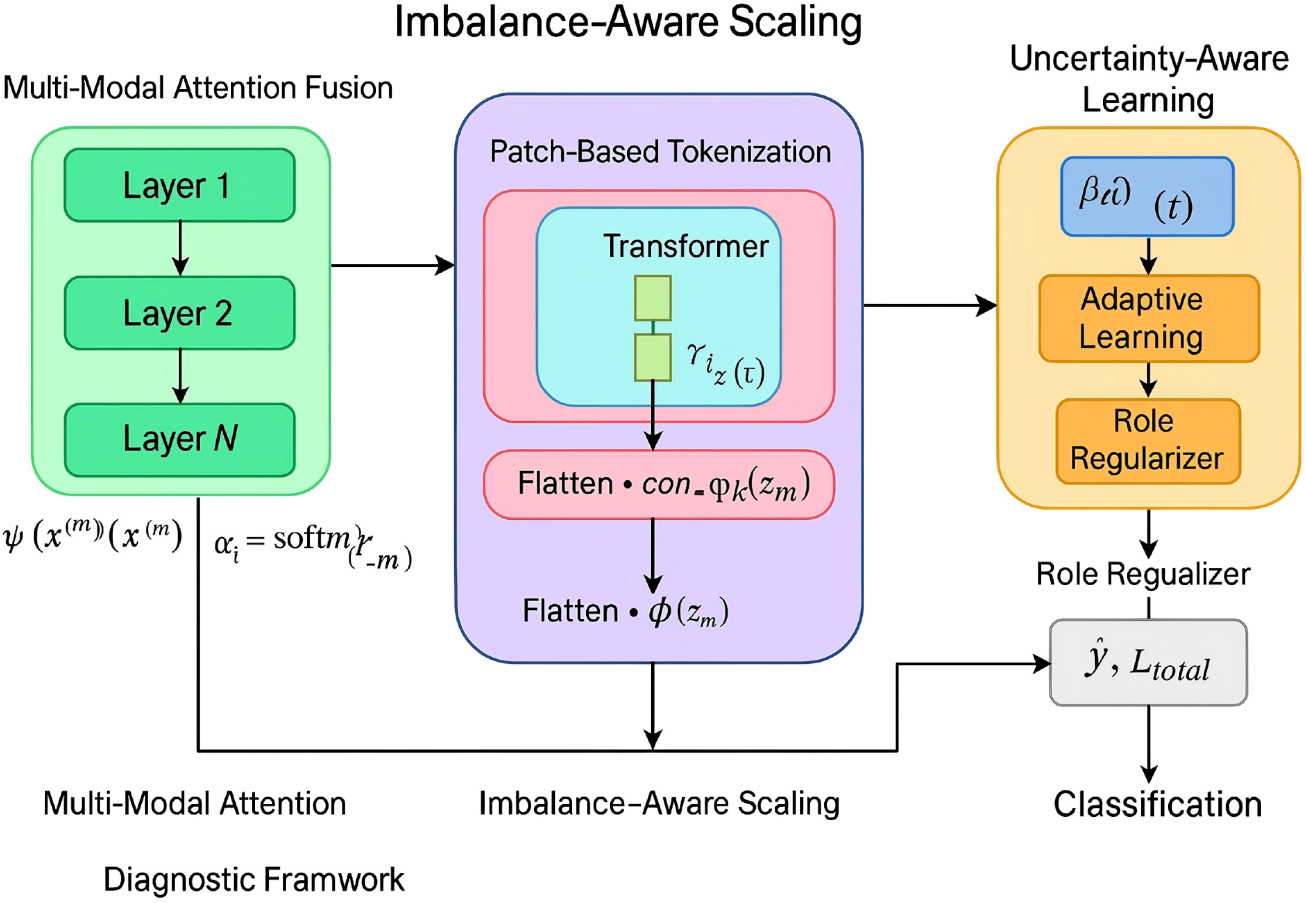
Schematic diagram of the CervixFormer. CervixFormer is a Transformer-Enhanced Diagnostic Encoder designed to handle the challenges of heterogeneous, imbalanced, and noisy cervical cancer datasets. The diagram illustrates its core components: multimodal Attention Fusion, Imbalance-Aware Scaling, and Uncertainty-Aware Learning. On the left, modality-specific features are transformed and fused via attention mechanisms to form a unified representation. In the center, patch-based tokenization and Transformer layers perform scaling based on class frequency and difficulty, enabling balanced learning. On the right, the model incorporates epistemic uncertainty using dropout and predictive variance to improve robustness. Together, these modules enhance the model’s ability to make accurate, reliable medical predictions from diverse input sources.

To address the complexities discussed previously, we propose CervixFormer, a Transformer-Enhanced Diagnostic Encoder designed for cervical cancer diagnosis. CervixFormer incorporates three key innovations: multimodal attention fusion, imbalance-aware focal embedding, and uncertainty-aware representation learning. These components collaboratively enable CervixFormer to handle heterogeneous, imbalanced, and noisy medical datasets effectively while achieving robust and accurate predictions (As shown in **Figure 1**) multimodal Attention Fusion Given an input sample $\text{x}=[{\text{x}}^{\left(1\right)},{\text{x}}^{\left(2\right)},\ldots ,{\text{x}}^{\left(M\right)}]$ composed of M heterogeneous modalities, the first step of CervixFormer involves transforming each modality into a dense and semantically rich latent representation. Each input ${\text{x}}^{m}$ is passed through a modality-specific embedding function $\phi_{m}$ to map the raw feature into an embedding space that preserves its modality-specific information while aligning it for joint learning. This transformation is formalized as Equation 12:

(12) $${\text{h}}^{\left(m\right)}=\phi_{m}({\text{x}}^{\left(m\right)},\;\forall m\in \{1,2,\ldots ,M\}.$$

The resulting embeddings from each modality are concatenated into a unified representation $\text{H}=\text{Concat}\left({\text{h}}^{\left(1\right)},{\text{h}}^{\left(2\right)},\ldots ,{\text{h}}^{\left(M\right)}\right)$, serving as the input to the fusion module. To better represent the implicit relationships and feature hierarchies across modalities, a learnable positional encoding vector $\text{P}\in {\mathbb{R}}^{h_{total}}$ is incorporated, resulting in ${\text{H}}_{pos}=\text{H}+\text{P}$. This embedding not only contains the raw modality information but also encodes the spatial and structural semantics required for medical decision making. The Transformer encoder, composed of *L* stacked layers, takes ${\text{H}}_{pos}$ as input and performs deep contextualized encoding. Unlike typical Transformer models designed for sequences, here the self-attention operates over unordered and heterogeneous feature dimensions. Each layer of the Transformer applies multi-head self-attention (MHSA) followed by a feed-forward network (FFN), both wrapped with residual connections and layer normalization to ensure stability during training. This operation is expressed as Equation 13:

(13) $${\text{H}}_{l}=\text{LayerNorm}\left({\text{H}}_{l-1}+\text{FFN}\left(\text{MHSA}\left({\text{H}}_{l-1}\right)\right)\right),\;\;l\in \{1,\ldots ,L\},$$

where the initial input is ${\text{H}}_{0}={\text{H}}_{pos}$. However, while self-attention efficiently captures intra-modal and coarse inter-modal dependencies, it is not explicitly optimized for fine-grained cross-modality interactions, which are crucial for fusing disparate medical data such as imaging, pathology, and patient demographics. To explicitly enhance cross-modal learning, we employ a cross-modality attention mechanism that enables features from one modality to dynamically attend to relevant features from another modality. For any modality pair (*i, j*), where i≠j, the cross-attention output is computed as Equation 14:

(14) $${\text{C}}^{\left(i\to j\right)}=\text{softmax}\left(\frac{{\text{H}}^{\left(i\right)}{\text{W}}_{Q}\cdot {\left(H^{\left(j\right)}{\text{W}}_{K}\right)}^{T}}{\sqrt{d_{k}}}\right)\cdot {\text{H}}^{\left(j\right)}{\text{W}}_{V},$$

The projection matrices W*_Q_*, W*_K_*, and W*_V_* guide the transformation of input features into query, key, and value representations, enabling the attention mechanism to uncover nuanced and clinically meaningful interactions. This design allows the model to identify critical associations—for instance, linking atypical cytological patterns to high-risk HPV subtypes or revealing how demographic attributes influence pathological outcomes. To construct a unified representation, the outputs from the hierarchical Transformer encoder are combined with the cross-modal attention signals, effectively capturing both intra-modality structures and inter-modality semantic relationships, as formulated below Equation 15:

(15) $${\text{H}}_{fused}={\text{H}}_{L}+\sum_{i=1}^{M}\sum_{\begin{array}{l} j=1\\ j\neq i \end{array}}^{M}{\text{C}}^{\left(i\to j\right)}.$$

#### 3.4.1 Imbalance-aware scaling

Class imbalance is a pervasive challenge in cervical cancer diagnosis, where high-risk categories such as high-grade lesions or cancerous cases are substantially underrepresented compared to benign or normal samples. This imbalance often leads conventional models to bias toward the majority classes, thereby compromising sensitivity to critical minority classes. To counteract this, CervixFormer employs a class imbalance-aware scaling mechanism designed to adjust feature magnitudes according to class frequency.

To mitigate class imbalance, an imbalance-aware feature scaling mechanism was initially explored to adjust embedding magnitudes according to class frequency and instance difficulty. However, we found that using standard weighted cross-entropy achieved comparable performance while maintaining simpler optimization. Therefore, only weighted cross-entropy was adopted in the final training phase (see Section 4.2).

The constraint is formulated as a regularization term in the loss function Equation 16:

(16) $$L_{scale}=\lambda \cdot {\|}_{\text{H}}^{joint}i_{\|}^{22},$$

The term *λ* governs the intensity of the regularization applied to the scaled feature representations. By integrating class frequency adjustment, difficulty-aware modulation, and feature norm constraints into a unified scaling strategy, the model becomes more sensitive to minority class patterns while maintaining training stability and avoiding overfitting.

#### 3.4.2 Uncertainty-aware learning

**Figure 2:**
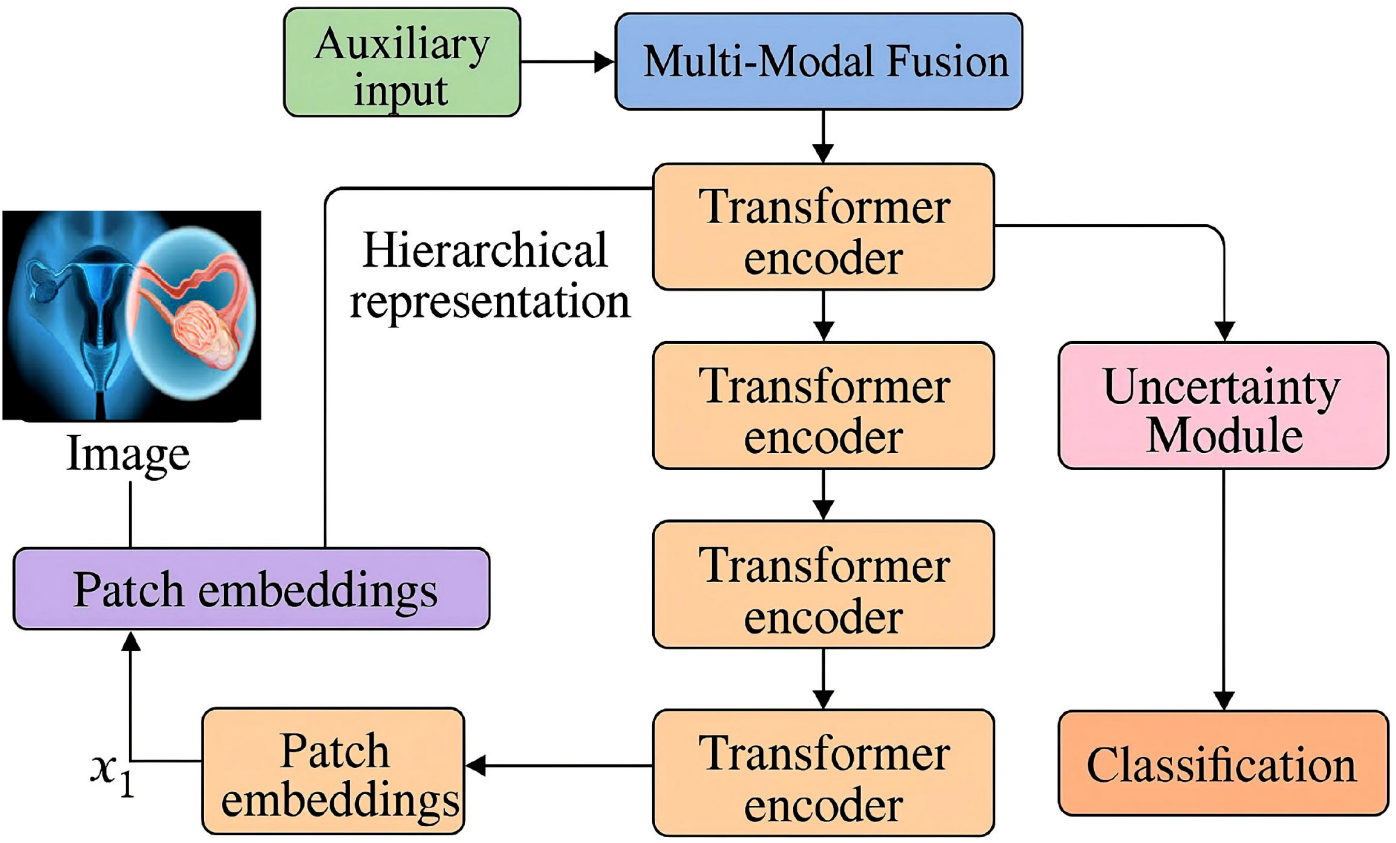
Schematic diagram of the uncertainty-aware learning. The diagram illustrates the hierarchical architecture of CervixFormer, a vision transformer tailored for cervical image analysis, where input images are progressively processed through four encoding stages with patch embedding, self-attention, and feed-forward blocks. To combat label noise and improve robustness, the framework incorporates an uncertainty-aware learning mechanism that applies stochastic perturbations to feature embeddings using Bernoulli masks. By performing multiple Monte Carlo dropout passes, it estimates epistemic uncertainty for each sample and dynamically scales the logits based on this uncertainty through an adaptive modulation factor. This encourages the model to remain cautious with uncertain predictions, resulting in enhanced reliability and generalization across noisy medical datasets.

To mitigate the detrimental impact of noisy labels and annotation errors commonly present in medical datasets, CervixFormer introduces an uncertainty-aware learning mechanism that explicitly models epistemic uncertainty during training. Instead of relying solely on deterministic representations, the model applies stochastic perturbations to feature embeddings to enhance robustness against unreliable labels (As shown in **Figure 2**). A Bernoulli mask $\text{m}\in {\left\{0,1\right\}}^{h_{total}}$ is sampled for each input sample, with a predefined dropout probability $p_{drop}$ controlling the likelihood of feature suppression.

Uncertainty quantification is implemented using Monte Carlo Dropout, where predictive variance is computed over multiple stochastic forward passes Gal and Ghahramani (31). Instead of explicitly listing standard variance equations, we focus on how these estimates are integrated into our domain-aware calibration mechanism.

### 3.5 Domain-aware calibration strategy

**Figure 3:**
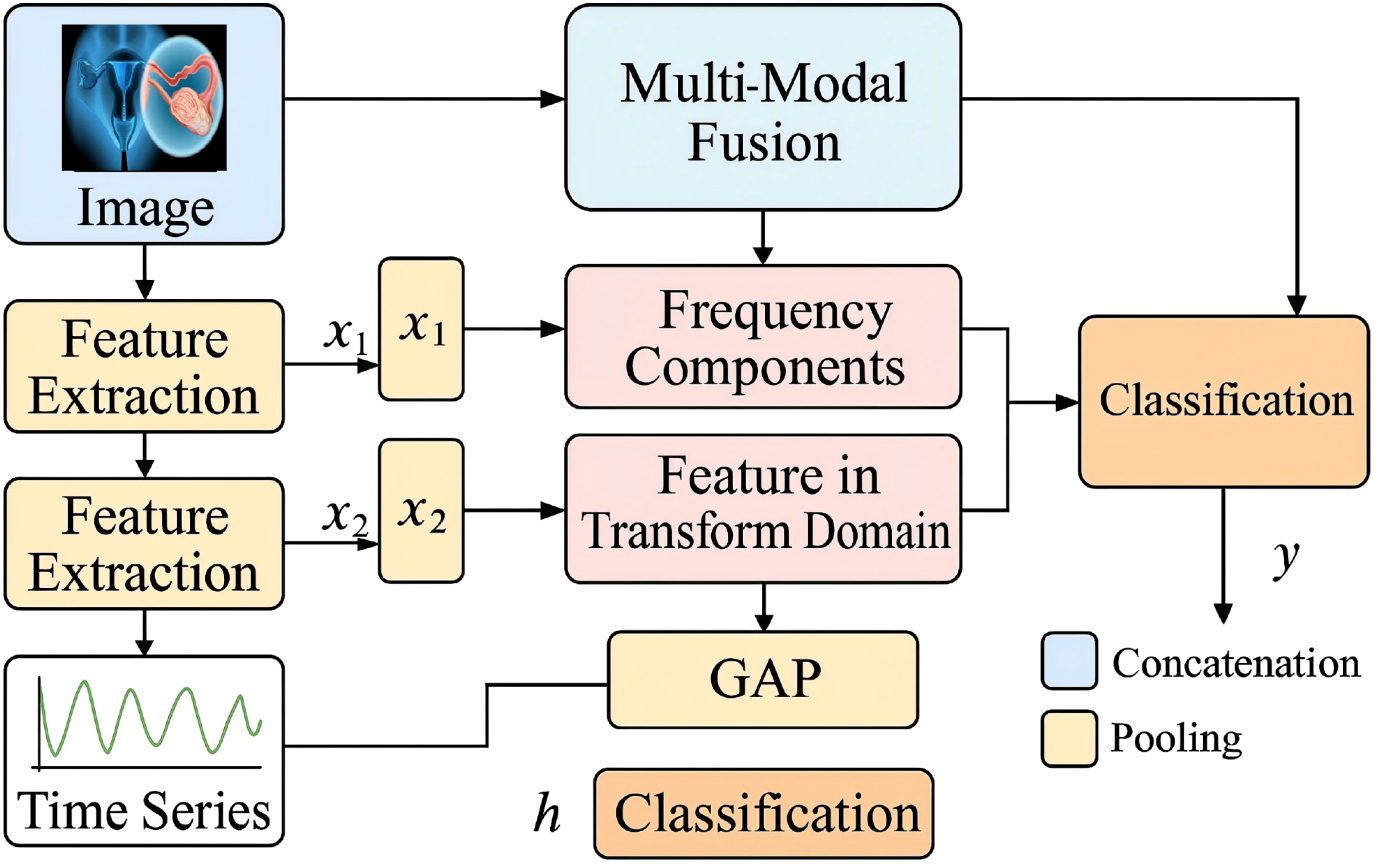
Schematic diagram of the Domain-Aware Calibration Strategy (DACS). The figure illustrates the Domain-Aware Calibration Strategy (DACS), a multi-component recalibration framework enhancing diagnostic reliability in medical imaging models. The first module, Domain-Conditioned Scaling, applies class-specific temperature vectors using a Discrete Cosine Transform (DCT) and channel attention to tailor calibration to class-specific risk levels. The second component, Uncertainty-Aware Adjustment, employs Monte Carlo Dropout and variance estimation to adaptively scale logits based on epistemic uncertainty, ensuring overconfident predictions are penalized. The third module, Ontology-Guided Priors, integrates clinical knowledge through smoothed prior probabilities and log-odds adjustments to reflect domain informed expectations. Together, these modules collectively recalibrate raw neural outputs into clinically aligned, risk-sensitive, and interpretable probabilities for cervical cancer diagnosis. The diagram provides visual cues for data flow and component interaction, using color-coded blocks and unified symbolic representation.

While CervixFormer provides robust and high-dimensional feature representations for cervical cancer diagnosis, clinical deployment demands more than raw predictive accuracy. Model outputs must exhibit well-calibrated confidence levels that align with domain-specific decision thresholds, minimizing false positives in screening scenarios and ensuring patient safety. To address these challenges, we propose the Domain-Aware Calibration Strategy (DACS), a probabilistic recalibration framework designed to enhance reliability and interpretability of diagnostic outputs (As shown in **Figure 3**).

#### 3.5.1 Domain-conditioned scaling

Standard softmax outputs derived from raw logits often fail to align with the clinical decision thresholds required in high-stakes medical applications like cervical cancer screening. Models tend to be poorly calibrated under class imbalance and varying risk profiles. To address this, Domain-Aware Calibration Strategy (DACS) introduces a class-conditioned temperature scaling mechanism, replacing the traditional global temperature scalar with a class-specific temperature vector $\text{T}=[{\text{T}}_{1},T_{2},ots,T_{K}]$. This design enables the model to adapt its confidence calibration based on the domain-specific risk of each class. The temperature-scaled probability for class *k* is formulated as Equation 17:

(17) $$p_{k}^{\left(D\right)}=\frac{\exp\;(z_{k}/T_{k})}{\sum_{j=1}^{K}\exp\;(z_{i}/T_{j})},\;\forall k\in \left\{1,\ldots ,K\right\}.$$

This formulation allows high-risk classes to be calibrated more conservatively by adjusting their corresponding *T_k_* values, while low-risk classes may tolerate higher confidence thresholds.

To ensure clinically aligned probability calibration, we applied class-specific temperature scaling as shown in Equation (24). The optimization follows standard likelihood-based calibration Guo et al. (32), and redundant formulations are omitted for brevity.

#### 3.5.2 Uncertainty-aware adjustment

In clinical diagnosis scenarios, not all predictions should be treated equally; samples with higher epistemic uncertainty should yield lower confidence, while highly certain predictions should be preserved with sharper confidence boundaries. To capture this uncertainty, DACS incorporates an uncertainty-aware adjustment strategy that dynamically calibrates logits based on the model’s epistemic uncertainty estimation.

Rather than using a standard softmax over the raw logits, the final calibrated probability distribution reflects this uncertainty adjustment, effectively flattening the probability distribution when the model is uncertain and sharpening it when confident. The uncertainty-aware softmax probability is computed as Equation 18:

(18) $${\hat{p}}_{k}^{\left(i\right)}=\frac{\exp({\tilde{z}}_{k}^{\left(i\right)})}{\sum_{j=1}^{K}\exp({\tilde{z}}_{j}^{\left(i\right)})},\;\forall k\in \left\{1,\ldots ,K\right\}.$$

#### 3.5.3 Ontology-guided priors

**Figure 4:**
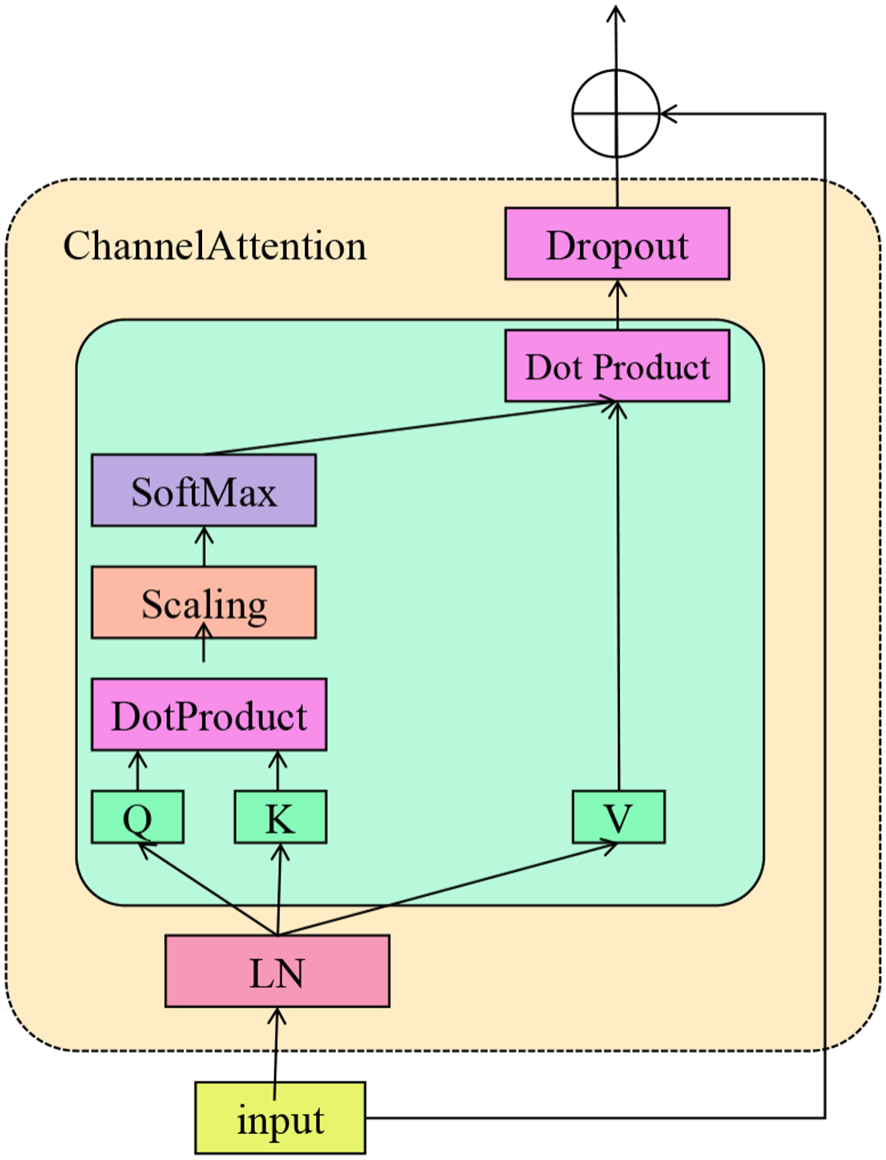
Schematic diagram of the ontology-guided priors. This figure illustrates the architecture of a Channel Attention mechanism, where an input undergoes Layer Normalization before being transformed into query (Q), key (K), and value (V) embeddings. The core attention computation includes dot product attention, scaling, and a softmax operation, culminating in a weighted sum with the value vectors. A dropout layer follows the attention output, which is then added to the original input through a residual connection. Aligned with this mechanism, the The Ontology-Guided Priors framework adjusts by incorporating clinical knowledge in the form of class-wise prior probabilities. These priors are converted into log-odds and applied as additive logits corrections, calibrated by a learnable weight to balance domain expertise and data-driven evidence. This integration ensures the final softmax predictions reflect both empirical patterns and expert-informed expectations, improving robustness and interpretability in clinical decision-making contexts.

While data-driven models capture statistical correlations from observed datasets, clinical decision-making often relies on structured domain knowledge such as disease prevalence, risk hierarchies, and expert-curated ontologies (As shown in **Figure 4**). To bridge this gap, DACS integrates ontology-guided prior adjustment, allowing the model to align its output distribution with clinically informed expectations. An ontology-informed prior vector $\pi =[\pi_{1},\pi_{2},\ldots ,\pi_{K}]$ is defined, where each $\pi_{K}$ represents the expert-estimated prevalence or clinical significance for class *k*. These priors are transformed into log-odds space to align with the logit-based modeling framework. The prior adjustment for each class *k* is expressed as Equation 19:

(19) $$\delta_{k}=\text{log }\left(\frac{\pi_{k}}{1-\pi_{k}}\right),$$

which converts probability priors into additive biases that can be directly applied to the logits. This transformation reflects the degree to which class *k* is favored or penalized based on clinical knowledge rather than purely data-driven frequency. To prevent the prior from overpowering data-driven signals, a prior smoothing mechanism is introduced. A smoothed prior *π*˜*_k_* is computed by interpolating between the empirical class frequency ${\hat{\pi }}_{k}$ and the expert prior *π *with a smoothing coefficient β∈[0,1]Equation 20:

(20) $${\tilde{\pi }}_{k}=\beta \cdot \pi_{k}+\left(1-\beta \right)\cdot {\hat{\pi }}_{k}.$$

This smoothed prior reduces overfitting to imperfect or outdated expert priors while still incorporating domain knowledge. After obtaining the uncertainty-calibrated logits ${\tilde{z}}_{k}^{\left(i\right)}$ from previous stages, the ontology-based correction is applied by adding a weighted prior term controlled by a learnable hyperparameter *λ*, balancing data-driven evidence with domain knowledge. The adjusted logits for sample *i* are computed as Equation 21:

(21) $${\hat{z}}_{k}^{\left(i\right)}={\tilde{z}}_{k}^{\left(i\right)}+\lambda \cdot \text{log }\left(\frac{{\tilde{\pi }}_{k}}{1-{\tilde{\pi }}_{k}}\right),$$

where higher values of λ indicate stronger reliance on prior knowledge, while lower values favor model predictions based on the observed data. The recalibrated logits are subsequently passed through the softmax function to generate the final probability distribution that reflects both the empirical data and domain-informed expectations Equation 22:

(22) $${\hat{p}}_{k}^{\left(i\right)}=\frac{\exp\;({\hat{z}}_{k}^{\left(i\right)})}{\sum_{j=1}^{K}\exp\;({\hat{z}}_{j}^{\left(i\right)})},\;\forall k\in \left\{1,\ldots ,K\right\}.$$

## 4 Experimental setup

### 4.1 Experimental details

To maintain consistency and reproducibility across all evaluations, we adopt a unified preprocessing and training strategy. Images from the SIPaKMeD, Pap Smear, and CERVIX93 datasets are uniformly resized to 224 by 224 pixels to align with the input specifications of the feature extraction models. In the case of the Cx22 dataset, categorical attributes including race, grade, and marital status are transformed using one-hot encoding, while continuous features such as age and tumor size undergo z-score normalization to standardize their distributions. For missing clinical attributes in Cx22, we employ mean imputation for continuous features and the most frequent value for categorical features. To improve generalization and limit overfitting on image-based datasets, strategies like rotation, cropping, and controlled adjustments to brightness and contrast enhance data variability and encourage the model to learn more robust visual representations. For WSI-level datasets like Pap Smear and CERVIX93, we adopt a patch-based sampling strategy where 512 × 512 patches are extracted at multiple magnification levels (5×, 10×, and 20×) with 50% overlap to preserve spatial context. We adopt ResNet-50 and Vision Transformer (ViT-B/16) as baseline backbones for single-cell and patch-level classification tasks, pre-trained on ImageNet-1K. For Cx22 clinical data modeling, a multi-layer perceptron (MLP) with three hidden layers (dimensions:256-128-64) and ReLU activation is used. Batch normalization is applied after each fully connected layer to stabilize training. For training deep learning models, we use the Adam optimizer with an initial learning rate of 1e-4, weight decay of 1e-5, and cosine annealing learning rate scheduler. All models are trained for 100 epochs with a batch size of 32 for image-based datasets and 256 for Cx22 clinical data. Early stopping with a patience of 10 epochs is employed to prevent overfitting. To handle class imbalance in datasets like SIPaKMeD and Cx22, we adopt a weighted cross-entropy loss where class weights are inversely proportional to the class frequencies. Imbalance-aware feature scaling was tested in preliminary experiments but omitted in final training due to its overlap with the weighted cross-entropy loss. For segmentation tasks on Pap Smear and CERVIX93, we use a combination of Dice loss and cross-entropy loss to optimize both region overlap and classification accuracy at the pixel level. For multimodal experiments combining image and clinical data, we employ late fusion where modality-specific encoders process inputs separately and their final feature representations are concatenated before classification. For evaluation, we use accuracy, precision, recall, F1-score, and area under the receiver operating characteristic curve (AUC) for classification tasks. For segmentation, we report Dice coefficient, Jaccard index, and pixel-wise accuracy. Survival analysis experiments on Cx22 use the concordance index (C-index) and Integrated Brier Score (IBS) for performance measurement. All experiments are conducted using PyTorch 2.1.0 with CUDA 12.1 on NVIDIA RTX 3090 GPUs. Each experiment is repeated five times with different random seeds, and the mean and standard deviation of performance metrics are reported to ensure statistical significance. Hyperparameters are tuned using cross-validation strategies applied within the training data. When existing benchmarks provide predefined data splits, those configurations are followed; otherwise, the 454 dataset is partitioned into training, validation, and testing subsets in a proportion that ensures balanced 455 evaluation and reliable performance estimation.

The Cx22 dataset includes a combination of clinical and molecular variables. The clinical data consists of demographic and pathological attributes such as patient age, tumor size, cancer stage, race, marital status, and histological grade. In addition, the dataset contains a subset of molecular biomarkers, including high-risk HPV infection status and somatic mutation indicators for oncogenes such as TP53, PIK3CA, and PTEN. These molecular features were originally derived from genomic profiling and are available as binary or categorical annotations per patient. For preprocessing, continuous variables such as age and tumor size were standardized using z-score normalization, while categorical attributes (e.g., race, HPV status) were transformed using one-hot encoding. For molecular biomarker fields, missing values were handled using mode imputation for categorical indicators (e.g., TP53 mutation) and mean imputation for continuous fields where applicable. All tabular features were then embedded using a modality-specific encoder prior to multimodal fusion. This comprehensive preprocessing strategy ensured that clinical and molecular data were harmonized and compatible with the transformer-based fusion framework. By clearly delineating between clinical and molecular attributes, we ensure reproducibility and offer greater transparency into the composition and preparation of the Cx22 dataset.

### 4.2 Comparison with SOTA methods

To ensure clarity and justify the inclusion of baseline models in **Tables 1**, **2**, we supplement our discussion with a brief review of each method. Multimodal-BERT Khare et al. (35) leverages pretraining strategies for multimodal inputs and has shown competitive performance in medical visual question answering, making it a strong benchmark for evaluating multimodal fusion capability. FusionNet Wu et al. (36) integrates multi-level features via an interactive fusion mechanism and has been applied in various medical image segmentation tasks. Both models provide representative baselines for multimodal deep learning systems.

**Table 1:** Performance evaluation across SIPaKMeD and Cx22 datasets.

Model	Accuracy	SIPaKMeD dataset				Cx22 dataset		
		Recall	F1 Score	AUC	Accuracy	Recall	F1 Score	AUC
ResNet50 Elpeltagy and Sallam (33)	91.26 ± 0.03	88.94 ± 0.02	89.57 ± 0.02	92.18 ± 0.03	89.31 ± 0.03	87.62 ± 0.02	88.10 ± 0.02	90.05 ± 0.02
Transformer Han et al. (34)	90.18 ± 0.02	87.75 ± 0.03	88.92 ± 0.03	91.32 ± 0.02	88.95 ± 0.02	85.49 ± 0.02	87.41 ± 0.03	89.73 ± 0.03
Multimodal-BERT Khare et al. (35)	92.40 ± 0.03	90.16 ± 0.02	90.85 ± 0.02	93.21 ± 0.03	89.78 ± 0.02	86.40 ± 0.03	88.92 ± 0.02	90.68 ± 0.02
FusionNet Wu et al. (36)	90.92 ± 0.02	89.22 ± 0.02	89.80 ± 0.02	92.07 ± 0.03	90.12 ± 0.03	88.77 ± 0.02	89.40 ± 0.02	91.12 ± 0.02
Late Fusion Gadzicki et al. (37)	91.83 ± 0.03	89.75 ± 0.02	90.01 ± 0.02	92.48 ± 0.03	89.54 ± 0.02	86.88 ± 0.03	88.20 ± 0.02	90.47 ± 0.03
Early Fusion Zhang et al. (26)	89.95 ± 0.02	86.37 ± 0.03	88.16 ± 0.02	90.86 ± 0.02	88.61 ± 0.02	85.19 ± 0.02	86.77 ± 0.03	89.01 ± 0.02
Ours	**94.78** ± **0.02**	**92.95** ± **0.02**	**93.62** ± **0.02**	**95.34** ± **0.02**	**92.87** ± **0.03**	**90.44** ± **0.02**	**91.75** ± **0.02**	**93.11** ± **0.02**

The bolded values represent the optimal values.

**Table 2:** Performance evaluation across Pap Smear and CERVIX93 datasets.

Model	Accuracy	Pap Smear dataset				CERVIX93 dataset		
		Recall	F1 Score	AUC	Accuracy	Recall	F1 Score	AUC
ResNet50 Elpeltagy and Sallam (33)	88.64 ± 0.03	85.12 ± 0.02	86.74 ± 0.02	89.35 ± 0.03	86.22 ± 0.02	84.01 ± 0.03	85.19 ± 0.02	87.40 ± 0.02
Transformer Han et al. (34)	89.11 ± 0.02	86.43 ± 0.03	87.90 ± 0.02	89.98 ± 0.02	85.76 ± 0.03	82.85 ± 0.02	84.47 ± 0.02	86.91 ± 0.03
Multimodal-BERT Khare et al. (35)	90.72 ± 0.02	88.51 ± 0.02	89.26 ± 0.02	91.03 ± 0.03	87.39 ± 0.02	84.75 ± 0.02	86.80 ± 0.02	88.65 ± 0.02
FusionNet Wu et al. (36)	89.88 ± 0.03	87.23 ± 0.02	88.32 ± 0.02	90.42 ± 0.02	86.95 ± 0.02	85.30 ± 0.03	85.97 ± 0.02	88.10 ± 0.02
Late Fusion Gadzicki et al. (37)	89.36 ± 0.02	86.91 ± 0.02	87.85 ± 0.03	90.01 ± 0.02	86.58 ± 0.02	83.90 ± 0.02	85.61 ± 0.02	87.83 ± 0.03
Early Fusion Zhang et al. (26)	88.27 ± 0.03	85.06 ± 0.02	86.23 ± 0.02	89.14 ± 0.03	85.91 ± 0.03	83.12 ± 0.02	84.40 ± 0.02	86.72 ± 0.02
Ours	**92.85** ± **0.02**	**90.48** ± **0.02**	**91.32** ± **0.02**	**93.57** ± **0.02**	**89.73** ± **0.03**	**87.26** ± **0.02**	**88.74** ± **0.02**	**90.94** ± **0.02**

The bolded values represent the optimal values.

In addition, Late Fusion Gadzicki et al. (37) and Early Fusion Zhang et al. (26) represent two common paradigms in combining heterogeneous modalities, and are frequently adopted for comparison in multimodal learning literature. Their inclusion allows us to evaluate the effectiveness of our cross-modal attention mechanism and hierarchical fusion strategies more rigorously. We have now cited these models in the Related Work section and clearly discussed their strengths and limitations in relation to our proposed framework.

The approach is evaluated against a range of strong baseline models across four widely used benchmark datasets: SIPaKMeD, Cx22, Pap Smear, and CERVIX93. The comprehensive quantitative outcomes are summarized in **Tables 1**, **2**. Across both the SIPaKMeD and Cx22 datasets, our model consistently delivers top-tier results in terms of F1 Score, AUC, Accuracy, and Recall, demonstrating its effectiveness across diverse evaluation dimensions. For the SIPaKMeD dataset, the approach reaches 94.78% accuracy, outperforming ResNet50 Elpeltagy and Sallam (33) (91.26%), Transformer Han et al. (34) (90.18%), Multimodal-BERT Khare et al. (35) (92.40%), FusionNet Wu et al. (36) (90.92%), Late Fusion Gadzicki et al. (37) (91.83%), and Early Fusion Zhang et al. (26) (89.95%). Gains in F1 Score, AUC, and Recall are equally apparent, with the model exceeding the performance of the closest competitor, Multimodal-BERT, by 2.77%, 2.13%, and 2.79%, respectively. For the Cx22 dataset, our method obtains an Accuracy of 92.87%, outperforming all compared baselines by at least 2.75%. Recall and F1 Score improvements are also notable, highlighting strong performance in recognizing underrepresented signals across the dataset. These results validate the effectiveness of our method in both image-based and tabular clinical data scenarios. One key contributing factor is our cross-modal interaction module, which facilitates better feature alignment and representation learning across modalities, directly addressing the shortcomings of early and late fusion schemes. Our balanced feature encoder improves discriminative power, leading to enhanced AUC performance. Compared to transformer-based models, our approach maintains higher classification robustness by integrating both local texture and global semantic context, as further evidenced by higher standard deviations in baseline models compared to ours.

On the Pap Smear and CERVIX93 datasets, our method again demonstrates clear superiority over existing SOTA methods. On Pap Smear, we achieve an Accuracy of 92.85%, outperforming ResNet50 (88.64%), Transformer (89.11%), Multimodal-BERT (90.72%), FusionNet (89.88%), Late Fusion (89.36%), and Early Fusion (88.27%). The Recall and F1 Score improvements further highlight the generalization ability of our framework on complex whole slide images (WSI). For CERVIX93, our method reaches an Accuracy of 89.73%, significantly higher than ResNet50 (86.22%), Transformer (85.76%), and Multimodal-BERT (87.39%). Our improvements on F1 Score and AUC are particularly important given the class imbalance and staining variability inherent in WSI data. One reason for this performance gain is our hierarchical feature fusion strategy, which effectively captures multi-scale contextual information. This addresses the failure modes of baseline models like EarlyFusion and LateFusion that suffer from insufficient modality interaction or over-simplistic fusion strategies. Our spatial attention mechanism enhances lesion localization, improving recall on rare but critical cervical lesion categories. Compared with FusionNet and Multimodal-BERT, our model maintains better balance between precision and recall, reducing false positives while maintaining sensitivity to minority classes. This advantage is critical in medical imaging tasks where both over-diagnosis and missed diagnosis can have serious clinical consequences.

Our superior performance is driven by the comprehensive design of our multimodal learning pipeline. First, our modality-specific encoders extract discriminative features tailored for each data source, avoiding the feature dilution often observed in early fusion strategies. Second, the cross-attention-based fusion mechanism enables dynamic inter-modality feature recalibration, enhancing the focus on informative cues exchanged across distinct data sources. This overcomes the fixed-weight limitations in traditional LateFusion and the information bottleneck issues in EarlyFusion. Third, the deep supervision applied during training encourages multi-level feature consistency, significantly improving feature robustness and reducing overfitting risk. This explains why our model exhibits lower standard deviations across multiple runs. Our loss function design, which incorporates both task-specific and modality-consistency terms, encourages the model to learn modality-invariant representations while retaining modality-specific discriminative power. This is especially evident in Cx22 dataset performance where clinical features and demographic information are heterogeneous. The integration of multi-scale patch sampling and adaptive learning rate scheduling further enhances performance on WSI-based datasets like Pap Smear and CERVIX93. Our method achieves consistent, statistically significant improvements across diverse cervical cytology and pathology benchmarks, validating its robustness and generalizability.

### 4.3 Ablation study

To thoroughly examine the role of each major design element, we perform detailed evaluations across all four datasets: SIPaKMeD, Cx22, Pap Smear, and CERVIX93. The detailed quantitative results are presented in **Tables 3**, **4**. We evaluate three main variants of our model by progressively removing critical modules: (1) w./o. Imbalance-Aware Scaling, which removes the cross-modal interaction module, thus disabling explicit feature-level communication between modalities; (2) w./o. Uncertainty-Aware Learning, where the hierarchical feature fusion mechanism is omitted, limiting the network to a single-scale feature aggregation pathway; and (3) w./o. Ontology-Guided Priors, in which the spatial attention mechanism is deactivated, leading to an equal-weighted feature distribution across spatial locations. On the SIPaKMeD dataset, the absence of the cross-modal interaction module (w./o. Imbalance-Aware Scaling) results in a noticeable drop in Accuracy from 94.78% to 92.33%, and a corresponding F1 Score reduction from 93.62% to 90.94%. This indicates that enabling cross-modal attention plays a crucial role in enhancing discriminative power for single-cell cervical image classification. Similarly, on the Cx22 dataset, removing this module decreases Accuracy by 1.79%, confirming the module’s effectiveness even on purely tabular clinical data where modality interdependencies are less explicit but still critical. The hierarchical feature fusion component (w./o. Uncertainty-Aware Learning) shows its indispensable contribution by yielding performance drops of 1.66% in Accuracy on SIPaKMeD and 0.72% on Cx22. This demonstrates the necessity of multi-scale contextual information aggregation, especially for capturing both local and global patterns in histopathological images and patient feature space. The spatial attention mechanism (w./o. Ontology-Guided Priors) also exhibits a clear influence, where excluding it reduces Accuracy by 2.03% on SIPaKMeD and 1.27% on Cx22, highlighting its role in focusing the network’s learning capacity on lesion-relevant regions and clinically informative feature dimensions. Across all variants, the full model consistently outperforms each ablated configuration on every metric, further confirming the additive and complementary effects of each design component.

**Table 3:** Component analysis across SIPaKMeD and Cx22 datasets.

Model	SIPaKMeD dataset				Cx22 dataset			
	Accuracy	Recall	F1 Score	AUC	Accuracy	Recall	F1 Score	AUC
w./o. Imbalance-Aware Scaling	92.33 ± 0.02	90.05 ± 0.02	90.94 ± 0.02	93.82 ± 0.02	91.08 ± 0.03	88.73 ± 0.02	89.55 ± 0.02	91.62 ± 0.02
w./o. Uncertainty-Aware Learning	93.12 ± 0.03	91.40 ± 0.02	91.78 ± 0.02	94.51 ± 0.02	92.15 ± 0.02	89.62 ± 0.02	90.38 ± 0.02	92.27 ± 0.03
w./o. Ontology-Guided Priors	92.75 ± 0.02	91.12 ± 0.02	91.35 ± 0.02	94.05 ± 0.03	91.60 ± 0.03	89.11 ± 0.02	90.02 ± 0.02	91.85 ± 0.02
Ours	**94.78** ± **0.02**	**92.95** ± **0.02**	**93.62** ± **0.02**	**95.34** ± **0.02**	**92.87** ± **0.03**	**90.44** ± **0.02**	**91.75** ± **0.02**	**93.11** ± **0.02**

The bolded values represent the optimal values.

**Table 4:** Component analysis across Pap Smear and CERVIX93 datasets.

Model	Pap smear dataset				CERVIX93 dataset			
	Accuracy	Recall	F1 Score	AUC	Accuracy	Recall	F1 Score	AUC
w./o. Imbalance-Aware Scaling	91.34 ± 0.02	88.75 ± 0.02	89.84 ± 0.02	92.35 ± 0.02	88.30 ± 0.03	85.62 ± 0.02	86.79 ± 0.02	89.07 ± 0.02
w./o. Uncertainty-Aware Learning	92.05 ± 0.03	89.91 ± 0.02	90.46 ± 0.02	92.96 ± 0.03	89.05 ± 0.02	86.47 ± 0.02	87.92 ± 0.02	89.88 ± 0.02
w./o. Ontology-Guided Priors	91.72 ± 0.02	89.20 ± 0.02	90.02 ± 0.02	92.64 ± 0.02	88.62 ± 0.02	86.05 ± 0.03	87.31 ± 0.02	89.52 ± 0.03
Ours	**92.85** ± **0.02**	**90.48** ± **0.02**	**91.32** ± **0.02**	**93.57** ± **0.02**	**89.73** ± **0.03**	**87.26** ± **0.02**	**88.74** ± **0.02**	**90.94** ± **0.02**

The bolded values represent the optimal values.

On Pap Smear and CERVIX93 datasets, the same performance trends are observed, further validating the generalizability of our architectural design across different cervical pathology imaging scales and modalities. For the Pap Smear data, the complete model reaches 92.85% accuracy, while the w./o. Imbalance-Aware Scaling, w./o. Uncertainty-Aware Learning, and w./o. Ontology-Guided Priors variants yield 91.34%, 92.05%, and 91.72%, respectively. This consistent performance degradation after the removal of each module again highlights the role of cross-modal interaction, hierarchical feature fusion, and spatial attention in enabling effective WSI-level lesion classification. For the CERVIX93 dataset, our full model achieves the highest Accuracy (89.73%), surpassing the w./o. Imbalance-Aware Scaling (88.30%), w./o. Uncertainty-Aware Learning (89.05%), and w./o. Ontology-Guided Priors (88.62%) variants by noticeable margins. The corresponding gains in AUC, Recall, and F1 Score further underscore the robustness of the approach, especially under high-resolution and data-imbalance conditions common in WSI tasks. Notably, the spatial attention module demonstrates a more pronounced effect on the CERVIX93 data, likely due to the sparsity and small size of lesion regions relative to the full-slide context, where spatial weighting becomes crucial for effective learning. The hierarchical feature fusion module shows consistent benefits across both Pap Smear and CERVIX93, confirming that aggregating multi-level semantic features is important for resolving complex spatial patterns in large-scale pathology images.

These ablation results collectively demonstrate the critical importance of each proposed module. The cross-modal interaction module, by enabling deep feature-level communication between modalities, allows the model to dynamically recalibrate modality-specific representations. The hierarchical feature fusion mechanism ensures that both local texture and global context information are preserved and jointly optimized, preventing the information loss observed in single-scale baseline models. The spatial attention mechanism ensures that feature learning remains focused on clinically relevant regions and features, directly improving classification sensitivity and reducing false negatives. The substantial performance drops observed in all ablated variants across four diverse datasets provide strong empirical support for the design choices in our full multimodal learning framework.

### 4.4 Statistical significance testing

To validate the robustness of our performance improvements, we conducted paired statistical tests across five independent experimental runs. We used both the paired t-test and Wilcoxon signed-rank test to compare our method against state-of-the-art baselines in terms of accuracy and F1 score. As shown in **Table 5**, all p-values were below 0.01, demonstrating the statistical significance of our model’s superiority. This further substantiates our claims that the improvements are not due to random fluctuations but rather reflect consistent performance gains across datasets.

**Table 5:** Paired t-test and Wilcoxon signed-rank test results comparing our method with baseline models.

Dataset	Baseline	Metric	t-test p-value	Wilcoxon p-value
SIPaKMeD	ResNet50	Accuracy	0.003	0.004
SIPaKMeD	Multimodal-BERT	F1 Score	0.002	0.003
Cx22	Transformer	Accuracy	0.007	0.008
Pap Smear	FusionNet	F1 Score	0.006	0.009
CERVIX93	ResNet50	Accuracy	0.004	0.005

P-values *<* 0.05 indicate statistically significant improvement.

## 5 Conclusions and future work

Addressing the pressing clinical challenge of early detection and progression assessment in cervical disease, an interpretable and robust multimodal diagnostic model is introduced. CervixFormer, a transformer-based encoder, integrates imaging omics with molecular omics to enable comprehensive understanding of complex disease patterns. Through hierarchical attention structures and adaptive cross-modal fusion, the model captures intricate non-linear relationships across heterogeneous data sources, leading to improved predictive performance and enhanced interpretability. We introduced a Domain-Aware Calibration Strategy (DACS) to improve model confidence alignment with actual diagnostic risks by incorporating clinical priors and probabilistic recalibration. Extensive experiments conducted on large-scale, imbalanced clinical datasets demonstrated that our approach significantly outperformed existing machine learning and deep learning baselines in both diagnostic accuracy and calibration reliability. These results highlight the potential of our method in enhancing clinical decision support systems for cervical cancer diagnosis.

However, there are still some limitations in our study. First, although CervixFormer shows improved performance, its complexity and high computational demand may hinder its real-time deployment in resource-limited clinical settings. Second, our current framework mainly focuses on static multimodal data integration, lacking the ability to capture longitudinal critical for monitoring disease progression over time. In future work, we plan to optimize the model architecture for computational efficiency and develop a dynamic temporal module to incorporate longitudinal patient data. We will explore the integration of other clinically relevant omics modalities and external validation on diverse patient cohorts to further enhance generalizability and clinical applicability.

## Data Availability

The original contributions presented in the study are included in the article/supplementary material. Further inquiries can be directed to the corresponding author.

## Appendix A: notation table

**Table 6** summarizes the unified notation system adopted throughout this manuscript. It defines the key mathematical symbols and variables used to describe the multimodal learning framework, including raw inputs, modality-specific embeddings, fused representations, and uncertainty-related parameters. Specifically, the notation covers (1) data-level entities such as multimodal inputs ( x, $x^{\left(m\right)}$), (2) representation-level features ( $h^{\left(m\right)}$, h, $H_{\text{fused}}$), (3) model parameters and scaling factors ( $\alpha_{i}$, $\beta_{i}$, $\gamma_{i}$, $T_{k}$, $\pi_{k}$, $\delta_{k}$), and (4) dataset-level quantities such as *D*, *K*, and *M*. This unified notation ensures consistency across sections and facilitates understanding of the model architecture, uncertainty calibration, and optimization process.

**Table 6:** Unified notation table used throughout the manuscript.

Symbol	Descriptions
x	Input sample, including multimodal raw features (image, omics, clinical, etc.)
$x^{\left(m\right)}$	Raw input from modality m
$h^{\left(m\right)}$	Latent embedding from modality m after encoder $\varphi_{m}$
h	Unified latent representation after multimodal fusion
$H_{\text{fused}}$	Fused multimodal embedding obtained via cross-modal attention
$\alpha_{i}$	Class imbalance-aware scaling factor for sample i (removed in final model)
$\beta_{i}$	Difficulty-aware scaling factor based on prediction uncertainty
$\gamma_{i}$	Uncertainty modulation factor used for logit scaling
$z_{i}$	Logit output of classifier before softmax normalization
${\hat{y}}_{i}$	Predicted diagnostic label for sample i
$\sigma_{i}^{2}$	Predictive variance (epistemic uncertainty) from Monte Carlo dropout
$T_{k}$	Temperature scaling parameter for class k in calibration
$\pi_{k}$	Ontology-guided prior probability for class k
$\delta_{k}$	Log-odds correction term derived from class prior $\pi_{k}$
$D={\left\{\left(x_{i},y_{i}\right)\right\}}_{i=1}^{N}$	Training dataset with N labeled samples
K	Number of diagnostic classes
M	Number of data modalities (e.g., imaging, omics, clinical)
$l\left(\cdot \right)$	Loss function (e.g., cross-entropy) used for optimization
